# The troglitazone derivative EP13 disrupts energy metabolism through respiratory chain complex I inhibition in breast cancer cells and potentiates the antiproliferative effect of glycolysis inhibitors

**DOI:** 10.1186/s12935-024-03319-z

**Published:** 2024-04-10

**Authors:** Claire Muller, Victorine Lacroix-Malgras, Jérôme Kluza, William Laine, Yonca Güler, Frédéric Bost, Michel Boisbrun, Sabine Mazerbourg, Stéphane Flament

**Affiliations:** 1 Université de Lorraine, CNRS, CRAN, F-54000 Nancy, France; 2 Université de Lille, CNRS, Inserm, CHU Lille, Institut Pour la Recherche Sur le Cancer de Lille, UMR 9020 – UMR-S 1277 - Canther – Cancer Heterogeneity, Plasticity and Resistance to Therapies, F-59000 Lille, France; 3 Inserm U1065, Université Côte d’Azur, Centre Méditerranéen de Médecine Moléculaire, Team Cancer Metabolism, Environment, F-06200, Nice, France; 4 Université de Lorraine, CNRS, L2CM, F-54000 Nancy, France; 5 CRAN, UMR 7039, Faculté des Sciences et Technologies, BP 70239, 54506 Vandœuvre-lès-Nancy, France

**Keywords:** Breast cancer, Thiazolidinediones, Troglitazone, Energy metabolism, Mitochondria, Glycolysis, Oxygen consumption

## Abstract

**Background:**

The metabolism of cancer cells generally differs from that of normal cells. Indeed, most cancer cells have a high rate of glycolysis, even at normal oxygen concentrations. These metabolic properties can potentially be exploited for therapeutic intervention. In this context, we have developed troglitazone derivatives to treat hormone-sensitive and triple-negative breast cancers, which currently lack therapeutic targets, have an aggressive phenotype, and often have a worse prognosis than other subtypes. Here, we studied the metabolic impact of the EP13 compound, a desulfured derivative of Δ2-troglitazone that we synthetized and is more potent than its parent compounds.

**Methods:**

EP13 was tested on two triple-negative breast cancer cell lines, MDA-MB-231 and Hs578T, and on the luminal cell line MCF-7. The oxygen consumption rate (OCR) of the treated cell lines, Hs578T mammospheres and isolated mitochondria was measured using the XFe24 Seahorse analyser. ROS production was quantified using the MitoSOX fluorescent probe. Glycolytic activity was evaluated through measurement of the extracellular acidification rate (ECAR), glucose consumption and lactate production in extracellular medium. The synergistic effect of EP13 with glycolysis inhibitors (oxamate and 2-deoxyglucose) on cell cytotoxicity was established using the Chou-Talalay method.

**Results:**

After exposure to EP13, we observed a decrease in the mitochondrial oxygen consumption rate in MCF7, MDA-MB-231 and Hs578T cells. EP13 also modified the maximal OCR of Hs578T spheroids. EP13 reduced the OCR through inhibition of respiratory chain complex I. After 24 h, ATP levels in EP13-treated cells were not altered compared with those in untreated cells, suggesting compensation by glycolysis activity, as shown by the increase in ECAR, the glucose consumption and lactate production. Finally, we performed co-treatments with EP13 and glycolysis inhibitors (oxamate and 2-DG) and observed that EP13 potentiated their cytotoxic effects.

**Conclusion:**

This study demonstrates that EP13 inhibits OXPHOS in breast cancer cells and potentiates the effect of glycolysis inhibitors.

**Supplementary Information:**

The online version contains supplementary material available at 10.1186/s12935-024-03319-z.

## Introduction

Breast cancer is the most frequently diagnosed cancer in women worldwide and in more developed regions of the world, this disease is the second cause of cancer-related death, after lung cancer [1]. Breast cancer treatments include surgery, radiotherapy, chemotherapy, endocrine therapy in case of hormone receptor-positive tumors and targeted therapies for tumors that overexpress human epidermal growth factor receptor 2 (HER2) [2]. However, approximately 25% of estrogen receptor-positive breast cancer patients do not respond to tamoxifen (anti-estrogen), and 50% of tamoxifen-treated patients develop a tamoxifen-resistant phenotype [3]. In addition, de novo and acquired resistance to trastuzumab (anti-HER2 monoclonal antibody) are also reported [4]. Finally, there are only a few therapeutics for triple-negative breast cancer (TNBC), an aggressive form of breast cancer characterized by the absence of estrogen, progesterone, and HER2 receptor expression [5, 6]. Indeed, two targeted treatments are now proposed: PARP inhibitors in patients having a germline BRCA1/2 mutation and immune checkpoint inhibition in tumors with PD-L1-positive immune cells [7]. Moreover, several antibody drug conjugates (ADCs) are under clinical investigation such as ladiratuzumab vedotin which presents encouraging safety and efficacy data [8]. To enhance clinical outcomes, several immune-based combinations are also being tested [9]. The combination of immune checkpoint inhibitor pembrolizumab plus ladiratuzumab vedotin is under evaluation in an ongoing phase Ib/II trial (NCT03310957) in TNBC with unresectable locally advanced or metastatic disease [10].

The increasing prevalence of drug-resistant cancers and the limited treatment efficacy for some subtypes such as TNBC are still strong arguments for the search for original therapeutic agents and optimized therapeutic strategies. In this context, the development of drugs able to target energy metabolism could be interesting since it is often affected in cancer cells. Indeed, cancer cells often exhibit a high rate of glycolysis and lactate production, even under normal oxygen concentrations, a phenomenon called the Warburg effect [11, 12]. In rapidly proliferating tumor cells, this specific metabolism responds to the necessity of rapid ATP generation, increased biosynthesis of macromolecules and maintenance of cellular redox homeostasis [11]. It is exploited by positon emission tomography (PET) imaging with 2-[^18^F] fluoro-2-deoxy-D-glucose and other glucose-based radiopharmaceuticals which allows tumor metabolic imaging [13].

Thiazolidinediones (TZD), a class of synthetic agonists of peroxisome proliferator-activated receptor gamma (PPARγ), initially used as insulin sensitizers for the treatment of type 2 diabetes, have been shown to affect cellular metabolism. For instance, troglitazone (TGZ) rapidly stimulated glycolysis in muscle tissue while reducing mitochondrial oxidative phosphorylation [14]. TGZ had a similar effect on glucose metabolism in mesangial cells [15]. Glitazones inhibited prostate cancer cells proliferation, which was accompanied by the stimulation of glycolysis and reduced oxygen consumption [16]. Breast cancer cells treated with TGZ showed an acute increase in glycolysis with reduced mitochondrial membrane potential [17].

The molecular mechanisms able to explain the anticancer effects of TGZ and related compounds have been extensively studied but they are not yet fully clarified. Clearly, PPARγ-independent mechanisms are involved, based on experiments using PPARγ antagonists, transfection of dominant-negative PPARγ isoforms, RNA interference targeting PPARγ as well as PPARγ-inactive compounds such as Δ2-TGZ or Δ2-ciglitazone (CGZ) [18]. These Δ2 analogs possess a double bond adjoining the thiazolidine-2,4-dione ring that creates structural rigidity, explaining the loss of PPARγ agonist activity [19]. In breast cancer cell lines, viability was reduced after exposure to Δ2-TGZ [20]. In the Δ2 family of compounds, we have shown that deoxygenated and substituted molecules had a higher antiproliferative action and a lower toxicity towards human hepatocytes [21, 22]. We recently reported that desulfuration led to a more potent molecule, EP13, which displayed an IC_50_ value of 5.8 µM in the estrogen-dependent MCF-7 cells and 5.9 and 8.8 µM in the triple-negative breast cancer cells MDA-MB-231 and Hs578T, respectively, in 2D cultures (compared to 74.3, 45.5 and > 100 µM for TGZ) [23]. In the present study, our aim was to determine whether EP13 could affect the energy metabolism of breast cancer cells and develop metabolic strategies to eradicate them.

## Materials and methods

### Chemicals

Compounds TGZ [22], Δ2-TGZ [22] and EP 13 [23] were prepared according to our previously reported procedures. Chemicals and reagents were purchased from Sigma‒Aldrich (Saint-Quentin Fallavier, France), unless otherwise stated.

### Cell culture

MDA-MB-231, Hs578T and MCF-7 human breast cancer cell lines were obtained from American Type Culture Collection (Manassas, VA, USA). These cells were grown at 37 °C under 5% CO_2_. Two culture media were used: Roswell Park Memorial Institute (RPMI) 1640 medium (Gibco®, Thermo Fisher Scientific, Villebon-sur-Yvette, France) for MDA-MB-231 and Dulbecco’s Modified Eagle Medium (DMEM, Gibco®) for Hs578T and MCF-7. Both of them were supplemented with 2 mM L-glutamine and 10% fetal bovine serum. In addition, in the case of Hs578T cells, DMEM was supplemented with 10 µg/mL insulin. Twenty-four hours after seeding, the cells were treated for various times with vehicle (DMSO) or different compounds at the indicated concentrations.

Hs578T spheroids were obtained by the liquid overlay technique. Ninety-six-well plates were coated with 1% agarose and seeded with 50 000 cells/well in DMEM supplemented with 2 mM L-glutamine, 10% fetal bovine serum and 10 µg/mL insulin. Hs578T cells formed one spheroid per well during the first day after seeding, and the spheroids were used for studies the second day after seeding the cells.

All cell lines were tested for mycoplasma contamination with a MycoAlert Mycoplasma Detection Kit (Lonza, Basel, Switzerland).

### Crystal violet staining assay

Cell numbers were determined after crystal violet staining (Sigma‒Aldrich, C6158) [23]. Cells were washed with phosphate-buffered saline (PBS), fixed and stained for 20 min with 0.2% crystal violet in 2% ethanol. In order to eliminate dye precipitates, the staining solution was filtered prior its use with a syringe-driven filter unit (0.22 µm pore size, Millipore, SLGP033RS). Cells were then washed with water to remove excess dye. Once dried, the dye was dissolved in 10% acetic acid. Cell number was determined by absorbance measurement at 595 nm with the multilabel plate reader VICTOR™ X3 (PerkinElmer, Courtaboeuf, France).

### Isolation of mitochondria

Mouse liver was homogenized in extemporaneously prepared isolation buffer (10 mM Tris/MOPS; 1 mM EGTA/Tris; 200 mM sucrose; pH 7.4) using a Dounce homogenizer. After centrifugation at 600 × g for 10 min at 4 °C, the collected supernatant was centrifuged at 7000 × g for 10 min at 4 °C. The pellet containing mitochondria was resuspended in 1 mL isolation buffer. Mitochondria were quantified using the DC protein assay (Bio-Rad, Marnes-la-Coquette, France) and resuspended at 10 μg/50 μL in extemporaneously prepared mitochondrial assay solution: 70 mM sucrose; 220 mM mannitol; 10 mM KH_2_PO_4_; 5 mM MgCl_2_; 2 mM HEPES; 1 mM EGTA; BSA Fatty acid free 0.2%; pH 7.4).

### Assessment of oxygen consumption

The oxygen consumption rate (OCR) was measured using the XFe24 Seahorse analyser (Seahorse Bioscience, Billerica, MA, USA) as previously published [24]. Briefly, the cells were seeded in 24-well plates (Seahorse XF24 V7 microplates) (Agilent France, Les Ulis) at 2.5 × 10^4^ cells/well. After 24 h, the cells were treated or not treated with different compounds. For 3D culture, one-day-old spheroids were transferred to Seahorse XFe96 Spheroid Microplates (Agilent France) and maintained in the complete culture medium as previously described. Spheroids were treated with increasing concentrations of EP13 or the vehicle DMSO for 4 h. At the end of the treatment, medium was removed and replaced by DMEM containing 2 mM L-glutamine, 10 mM D-glucose and 1 mM pyruvate. The cells or the spheroids were incubated for 30 min at 37 °C, and the following drugs were added: 1 μM oligomycin, 0.25–0.5 μM FCCP (carbonyl cyanide-p-trifluoromethoxyphenyl-hydrazon), 1 μM rotenone and 1 μM antimycin A.

To measure a direct effect of EP13 on MDA-MB-231 during a time-course of OCR measurement, EP13 (1, 3, 6 or 12 μM) or rotenone (1 μM) were injected in each well at the indicated times. OCR was measured every 7 min using XFe24 Seahorse. At the end of the experiment, a high dose of rotenone (6 μM) was injected to abolish oxygen consumption.

The mitochondrial suspension was loaded in a 24-well plate (Seahorse XF24 V7 microplates) (Agilent France) at 50 μL/well. The plate was centrifuged at 2000 g for 20 min at 4 °C. Then, mitochondria were incubated for 10 min with or without EP13 in 450 μL of mitochondrial assay solution at 37 °C, supplemented differently according to the protocol used: pyruvate (10 mM), glutamate (10 mM) and malate (10 mM) or succinate (10 mM) and rotenone (2 μM). Then, activators/inhibitors were added: ADP (5 mM), antimycine A (4 μM), ascorbate (10 mM), TMPD (N,N,N,N’-tetramethyl-p-phenylenediamine) (100 μM).

### Assessment of extracellular acidification rate

The extracellular acidification rate (ECAR) was measured using the XFe24 Seahorse analyser (Seahorse Bioscience, Billerica, MA, USA) as previously published [24]. Briefly, the cells were seeded in 24-well plates (Seahorse XF24 V7 microplates) (Agilent France, Les Ulis) at 2.5 × 10^4^ cells/well. After 24 h, the cells were treated with EP13 (6 µM) or the vehicle DMSO for 4 h. At the end of the treatment, medium was removed and replaced by DMEM containing L-glutamine (2 mM) and NaCl (32 mM). The cells were incubated for 30 min at 37 °C, and the following  compounds were added: 10 mM D-glucose, 1 µM oligomycin A and 100 mM 2-deoxyglucose.

### Measurement of ROS (reactive oxygen species) production

MDA-MB-231 and MCF-7 cells were plated in 60 mm cell culture dishes at 8 × 10^5^ and 6.5 × 10^5^ cells per dish. Twenty-four hours later, the cells were treated for 4 or 24 h with 6 µM EP13. Control cells were treated with DMSO (0.012%). At the end of the treatment, cells were incubated in the presence of 5 μM MitoSOX™ mitochondrial superoxide indicator fluorescent probe (Thermo Fisher Scientific) for 30 min at 37 °C protected from light. Once washed with PBS, the cells were resuspended in culture medium after trypsin incubation and counted using the TC20™ Automated Cell Counter (Bio-Rad). After centrifugation (200 g, 5 min) at room temperature, the cells were resuspended in culture medium at 1 × 10^6^ cells/mL. A total of 10^4^ cells were analysed, and the intensity of fluorescence indicative of superoxide anion production was determined by flow cytometry (BD FACSCaliburTM flow cytometer, BD Biosciences). Data were analysed with CellQuest™ Pro (BD Biosciences) software.

### Measurement of the effect of NAC (N-acetyl-L-cysteine) on cell number

Cells were seeded in 96-well plates at 1.5 × 10^4^ cells per well. Twenty-four hours later, the cells were pretreated with 1 mM NAC for 30 min, followed by the addition of H_2_O_2_ (50 µM), EP13 (6 µM) or vehicle (DMSO). The stock solution of 100 mM NAC was prepared extemporarily in DMEM supplemented with 1 N NaOH. After 48 h of treatment, the number of cells was determined by crystal violet staining assay as previously described.

### Measurement of glucose and lactate concentrations in extracellular medium

Glucose and lactate concentrations in culture media were measured using a YSI 2950 Biochemistry Analyser (YSI Life Sciences, Yellow Springs, OH, USA). Lactate and glucose concentrations were calculated by subtracting the blank value (medium without cultured cells) from the value measured for each condition. The values were normalized to the cell numbers evaluated by the crystal violet staining assay.

### Measurement of the combination index and isobologram representation

Cells were seeded in 96-well plates at 1.5 × 10^4^ cells per well. Twenty-four hours later, the cells were treated for 72 h with EP13 (3 or 6 µM) and increasing concentrations of oxamate, used alone or in combination. At the end of the treatment, the number of cells was determined by crystal violet staining assay as previously described [23]. The percentage of cell growth inhibition was then determined for each concentration of compound. Data were analysed using CompuSyn software (CompuSyn Version 1.0 by Ting Chao Chou and Nick Martin, 2004) [25, 26]. Combination index (CI) values were determined to assess the nature of compound-compound interactions that can be additive (0.9 < CI < 1.1), synergistic (CI < 0.9), or antagonistic (CI > 1.1) for various concentrations.

To study the EP13 and 2-deoxy-D-glucose (2-DG) combination, each compound was used either alone or in combination in a dose-dependent manner with a fixed ratio of 1/1. CI values were determined as described above. To further characterize the dose-dependent interaction of EP13 and 2-DG, isobolograms at effect levels of 25%, 50%, and 75% cell number decrease (Fa, fraction affected) were created using CompuSyn software. In each of these conditions, the dose requirements (IC_25_, IC_50_, IC_75_) were determined for each drug in combination or in single use. Data points from combination treatment above or below the line of additivity indicate antagonism or synergy, respectively.

### Statistical analysis

The results are depicted as the mean ± standard error of the mean (SEM) or standard deviation (SD) of at least three independent experiments. Statistical analyses were performed using Student’s t test or one-way analysis of variance (ANOVA) followed by Tukey’s multiple comparisons post-test, unless mentioned otherwise (GraphPad InStat software, San Diego, CA, USA). Differences in which the P value was less than 0.05 were statistically significant.

## Results

### EP13 treatment induces early OXPHOS inhibition in breast cancer cell lines.

**Fig. 1:**
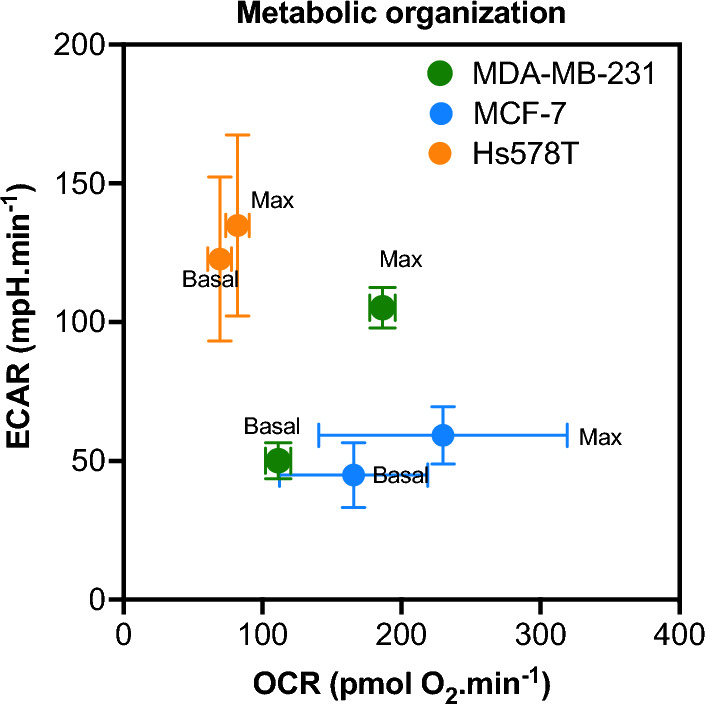
Metabolic profiles of MCF-7, MDA-MB-231 and Hs578T cells. Mitochondrial oxidative phosphorylation and glycolysis were assessed using XFe24 Seahorse to measure basal OCR (untreated condition) and maximal OCR (after oligomycin and FCCP treatment) and basal ECAR (untreated condition) and maximal ECAR (after oligomycin treatment). The results are depicted as the mean ± S.D. of at least three independent measurements

First, we determined the metabolic profile of the three breast cancer cell lines used in this study. Our analyses showed that the basal and maximal OCR (mitochondrial oxygen consumption rate) were very different with  MCF-7 > MDA-MB-231 > Hs578T cells (Fig. 1). The three cell lines also displayed different basal and maximal ECAR values (which mainly reflect glycolysis) with Hs578T > MDA-MB-231 and MCF-7 (Fig. 1).

**Fig. 2:**
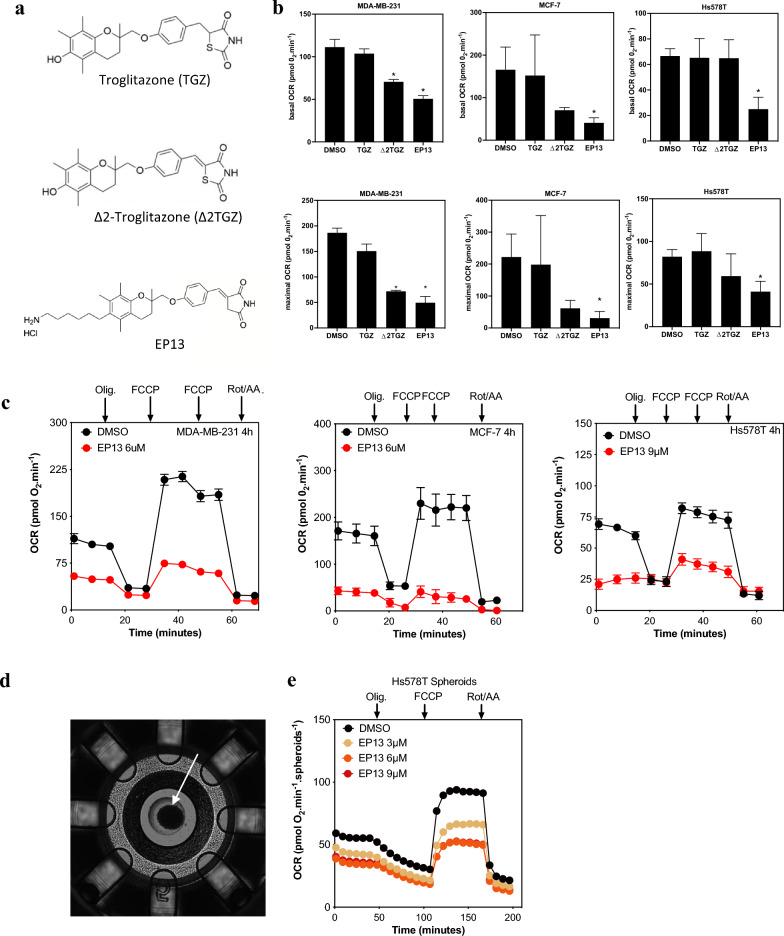
EP13 inhibits mitochondrial respiration in MDA-MB-231, MCF-7, and Hs578T cells. **a** Chemical structures of TGZ, Δ2-TGZ, and EP13. **b** MDA-MB-231, MCF-7, and Hs578T cells were grown in 2D conditions and treated for 4 h with vehicle, TGZ (45 µM, 74 µM and 50 µM, respectively), Δ2-TGZ (60 µM, 60 µM and 50 µM, respectively) or EP13 (6 μM, 6 µM and 9 µM, respectively), and the OCR was measured using XFe24 Seahorse. Basal and maximal respiration are depicted as the mean ± S.D. of at least three independent measurements (*, p < 0.05). **c** Assessment of the OXPHOS parameters of the breast cancer cell lines. At the times indicated, the following drugs were injected: oligomycin A (Oligo; 2 µM), FCCP (1.1 µM and 2.2 µM), and rotenone/antimycin A (Rot/AA; 1 µM each). Data are the mean ± S.D. (at least n = 3 wells per group). **d** A typical Hs578T spheroid observed before the measurement of OXPHOS parameters. **e** Assessment of the OXPHOS parameters on Hs578T spheroids exposed to increasing concentrations of EP13. The results are depicted as the mean ± S.D. (at least n = 3 wells per group)

Then, we studied the metabolic effects of EP13 versus its parent compounds Δ2-TGZ and TGZ using oximetry approaches (Fig. 2). The structures of the three molecules are shown in Fig. 2a. TGZ and Δ2-TGZ were used at their IC_50_ concentrations, except for the Hs578T cell line. With this cell line, the concentration used was 50 µM since the IC_50_ was superior to 100 µM [23]. After 4 h of TGZ exposure, we did not observe any significant change in the basal and maximal OCRs, regardless of the cell line (Fig. 2b). Δ2-TGZ decreased the basal OCR of MDA-MB-231 cells, and we also observed a high tendency to decrease OCR in MCF-7 cells (Fig. 2b). Under the same conditions, EP13 strongly decreased basal respiration in MDA-MB-231, MCF-7 and Hs578T cells (Fig. 2b). In these three cell lines, EP13 treatment also decreased the FCCP-stimulated OCR (maximal OCR) (Fig. 2b and 2c). Thus, EP13 clearly inhibited mitochondrial respiration in the triple-negative cell lines MDA-MB-231 and Hs578T, as well as in the luminal subtype cell line MCF-7. EP13 was also tested on spheroids of breast cancer cells. In our hands, only the triple-negative Hs578T cells easily developed spheroids (Fig. 2d). We observed that EP13 decreased the basal and maximal OCR in this 3D model, with effects detected at concentrations as low as 3 µM (Fig. 2e).

### EP13 directly targets isolated mitochondria and inhibits complex I of the respiratory chain

**Fig. 3:**
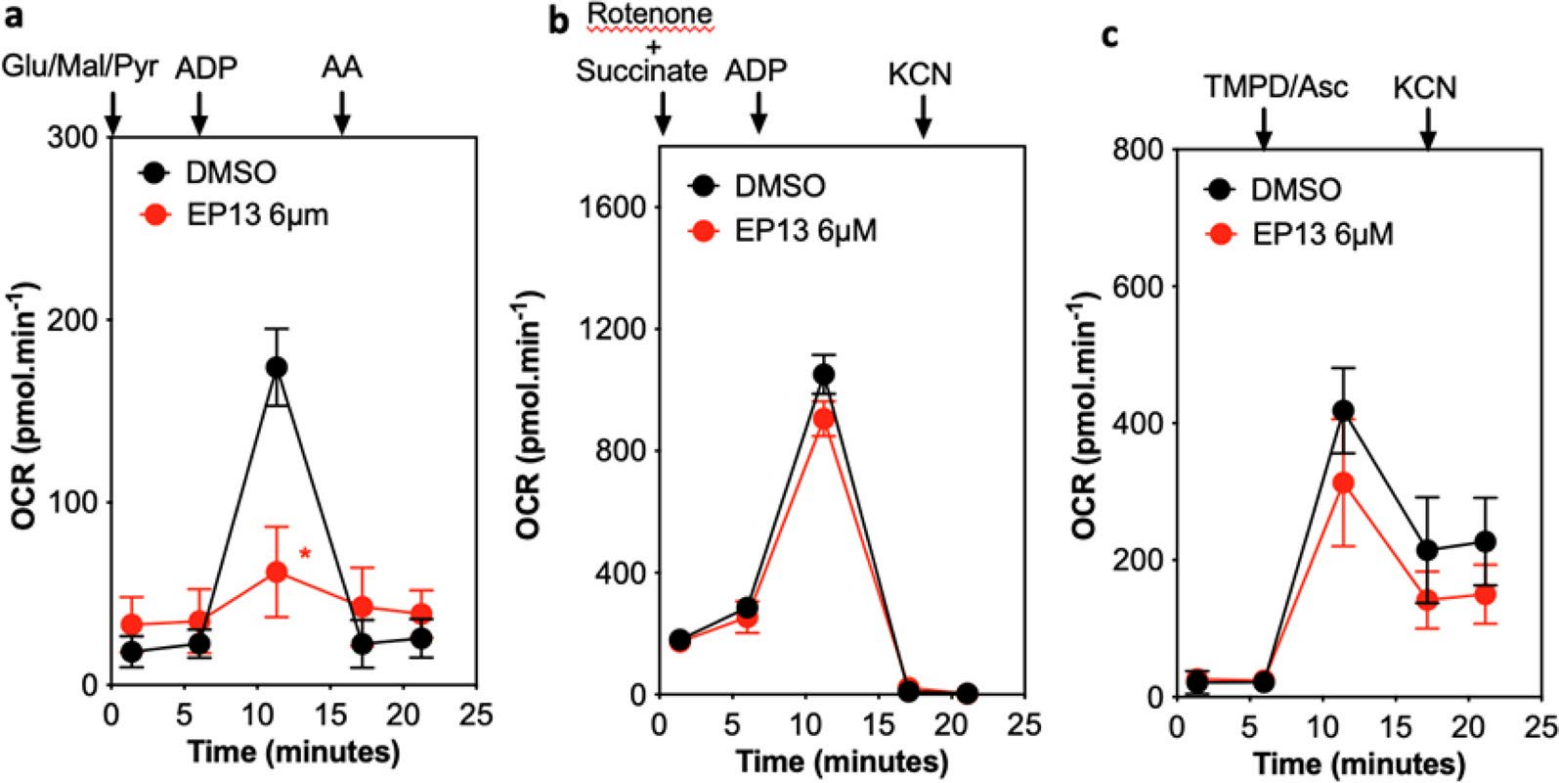
EP13 inhibits complex I but not complexes II, III, and IV of the respiratory chain. Mitochondria isolated from mouse liver were incubated for 10 min with EP13 (6 μM). The rate of oxygen consumption was measured using XFe24 Seahorse. **a** Addition of glutamate (Glu, 10 mM); malate (Mal, 10 mM) and pyruvate (Pyr, 10 mM) allowed the complex I activity; **b** Addition of rotenone (Rot, 2 µM) and succinate (10 mM) enabled the inhibition of the complex I and the functioning of the complex II, respectively; **c** Addition of TMPD (N,N,N′,N′-tetramethyl-p-phenylenediamine; 100 µM) and ascorbate (Asc, 10 mM) permitted the complex IV activity. Adenosine diphosphate (ADP, 5 mM) was used to stimulate oxidative phosphorylation. Antimycin A (AA, 4 µM) was used as a complex III inhibitor. Potassium cyanide (KCN) was used to block mitochondrial respiration. Data are the mean ± S.D. (at least n = 3 wells per group) (*, p < 0.05)

To determine whether EP13 could directly target mitochondria, we measured oxygen consumption in mitochondria isolated from mouse liver (Fig. 3). First, we specifically studied oxygen consumption related to NADH dehydrogenase complex I. Mitochondria were incubated with glutamate, malate, and pyruvate, which provide NADH for respiratory chain activity, and ADP was used to stimulate oxidative phosphorylation. Figure 3a shows that complex I-related oxygen consumption was inhibited when mitochondria were treated with EP13 compared with the control. Indeed, the addition of the ubiquinol-cytochrome c reductase complex III inhibitor (antimycin A) decreased respiration in controls but not in EP13-treated mitochondria since respiration was already inhibited. Next, we specifically studied succinate dehydrogenase complex II-related oxygen consumption (Fig. 3b). Mitochondria were incubated with rotenone, an inhibitor of complex I, and succinate, which provides FADH2, allowing complex II operation. In isolated mitochondria exposed to EP13, the OCR related to complex II was similar to that of control mitochondria. KCN strongly reduced respiration in EP13-treated mitochondria and control mitochondria. Finally, we studied cytochrome c oxidase complex IV (Fig. 3c). TMPD is a complex IV-specific electron donor, while ascorbate ensures that the TMPD is reduced and continues to donate electrons to build a linear rate of complex IV activity. Mitochondrial respiration is maintained in both control and EP13-treated cells, indicating that complex IV functions in both conditions, while KCN strongly reduced respiration in both cases.

**Fig. 4:**
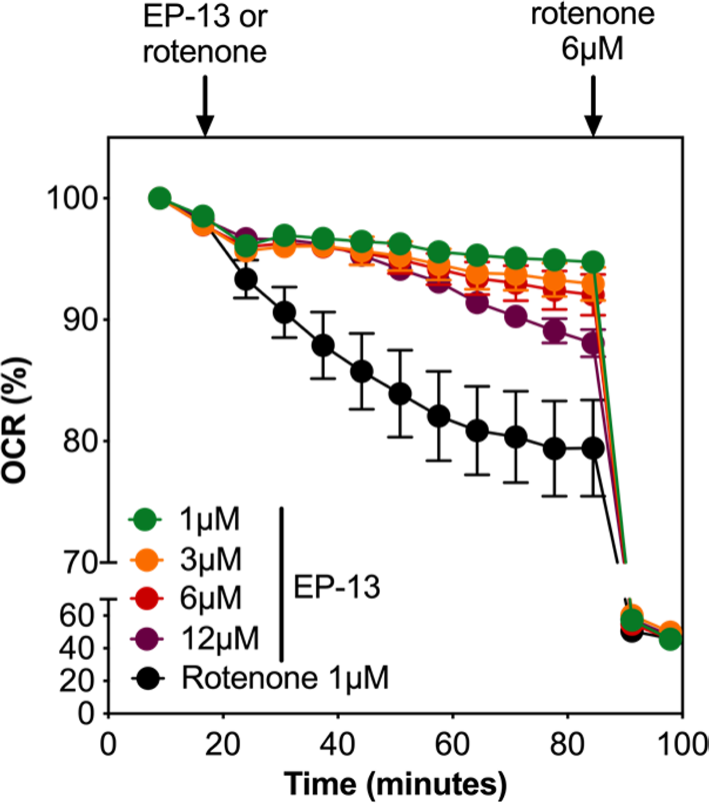
EP13 shows a rotenone-like activity. MDA-MB-231 cells were grown in 2D conditions and treated with EP13 (1, 3, 6 or 12 µM) or the complex I inhibitor, rotenone (1 µM) during a time-course (every 5 min) of OCR measurement using XFe24 Seahorse. At the end of the experiment, addition of 6 µM rotenone in both conditions completely blocked the oxygen consumption. Data are the mean ± S.D. (at least n = 3 wells per group)

To comfort our result on the inhibitory activity of EP13 on NADH dehydrogenase complex I, we compared EP13 with the complex I inhibitor rotenone on MDA-MB-231 OCR (Fig. 4). After addition of EP13 during a time-course of OCR measurement, the oxygen consumption decreased gradually with time, in a dose-dependent manner. Indeed, 12 µM EP13 induced a slight decrease of oxygen consumption after 7 min followed by a more pronounced effect after 50 min. The decrease reached 12.3% at 90 min. In the same condition, addition of rotenone (1 µM) led also to a time-dependent decrease of OCR that reached 19.6% at 90 min. Although EP13 showed a slower response compared to rotenone, this result was in agreement with a direct inhibitory effect on the complex I of the respiratory chain.

### Mitochondrial dysfunction induced by EP13 is associated with ROS production

**Fig. 5:**
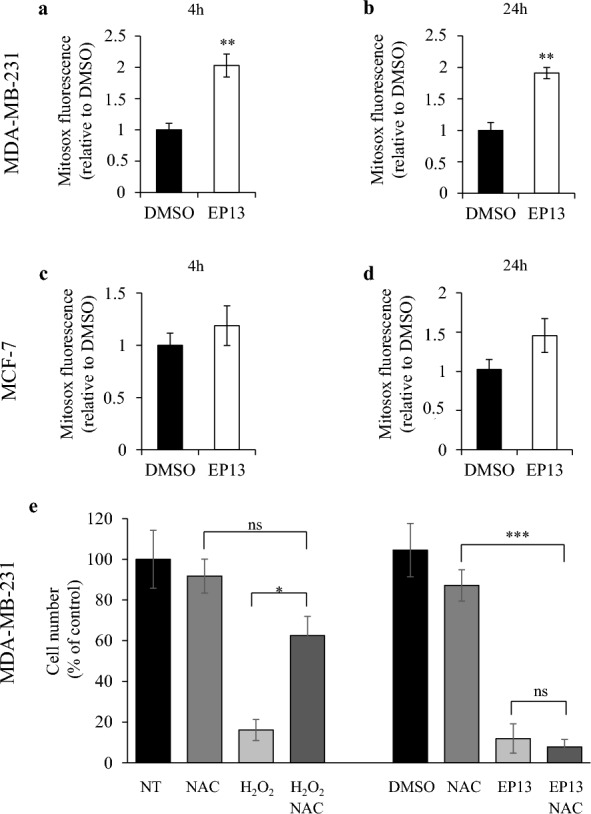
Mitochondrial ROS in EP13-treated cells. MDA-MB-231 (**a**, **b**) and MCF-7 (**c**, **d**) cells were treated for 4 or 24 h with vehicle or EP13 (6 μM). At the end of treatment, the cells were incubated in the presence of 5 µM MitoSOX™ Red fluorescent probe and analysed by flow cytometry. **e** MDA-MB-231 cells were treated for 48 h with H_2_O_2_ (50 µM) or EP13 (6 µM) in the presence or absence of the antioxidant NAC (1 mM). The results are depicted as the mean ± SEM of at least three independent measurements. Significant differences from control cells are indicated (*, p < 0.05; **, p < 0.01; ns, non significant)

We then studied mitochondrial ROS production since it is related to respiratory chain dysfunction. We measured superoxide anion levels by flow cytometry using the MitoSOX Red™ mitochondrial superoxide fluorescent indicator. After 4 h of EP13 treatment, we observed, as expected, an increase in ROS in MDA-MB-231 cells, which was approximately twofold higher than that in control cells (Fig. 5a). This ROS increase was still observed after 24 h of treatment with EP13 (Fig. 5b). In MCF-7 cells treated with EP13, despite a tendency, we did not observe a significant increase in ROS in comparison to control cells after 4 and 24 h (Fig. 5c and d). To determine whether the increase in ROS was involved in the effect of EP13, we used the antioxidant NAC (Fig. 5e). We observed that the presence of NAC inhibited the effect of H_2_O_2,_ but NAC was not sufficient to suppress the anti-cancerous effect of EP13, suggesting that its lethal action is mainly mediated by energetic stress.

### Increased glycolytic activity supports ATP production upon inhibition of OXPHOS by EP13

**Fig. 6:**
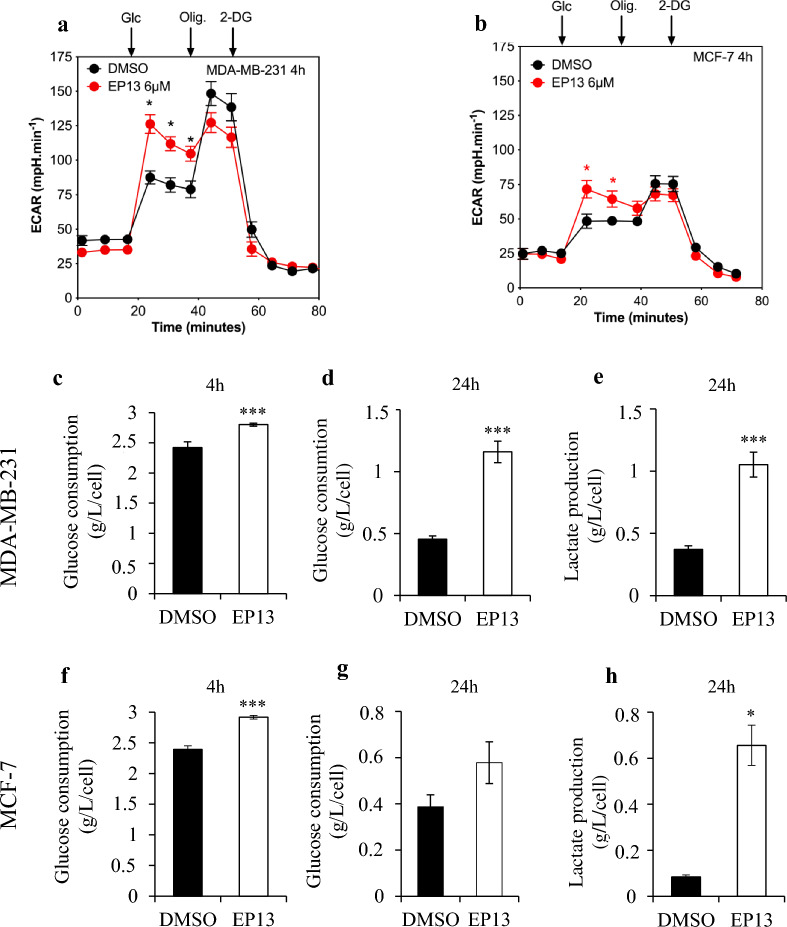
EP13 increases the extracellular acidification rate, glucose consumption and lactate production in MDA-MB-231 and MCF-7 cells. MDA-MB-231 (**a**, **c**, **d**, **e**) and MCF-7 (**b**, **f**, **g**, **h**) cells were treated for 4 h (a, b, c, f) or 24 h (d, e, g, h) with vehicle or EP13 (6 μM). The extracellular acidification rate (ECAR) (**a**, **b**) was measured using XFe24 Seahorse. At the indicated times, the following drugs were injected: Glc (D-glucose; 10 mM), oligomycin A (Oli; 1 µM) and 2DG (2-deoxyglucose 100 mM). The results are depicted as the mean ± S.D. (at least n = 3 wells per group). Glucose (**c**, **d**, **f**, **g**) and lactate (**e**, **h**) concentrations (g/L) were measured in culture medium and normalized relative to cell numbers. The results are depicted as the mean ± SEM of at least three independent measurements. Significant differences from control cells are indicated (*, p < 0.5; ***, p < 0.001)

After assessing mitochondrial energy metabolism, we investigated the effect of EP13 on glycolytic activity by measuring the extracellular acidification rate, glucose consumption and lactate production in MDA-MB-231 and MCF-7 cells. After 4 h treatment with EP13, we observed an increase of 21.06% and 45.33% in the extracellular acidification rate in MCF-7 and MDA-MB-231 cells, respectively, after addition of glucose (Fig. 6a and 6b). To support the hypothesis of an increase of the glycolytic activity of the cells, glucose consumption and lactate production were measured. After 4 h of EP13 exposure, a slight increase in glucose consumption was observed in MDA-MB-231 and MCF-7 cells (Fig. 6c and f). After 24 h of exposure to EP13, glucose consumption and lactate production increased 2.4-fold and 2.7-fold, respectively, in MDA-MB-231 cells (Fig. 6d, e). Similar results were observed in MCF-7 cells (Fig. 6g and h). The increase in glycolysis could compensate for the inhibition of oxidative phosphorylation to maintain sufficient energy in the cells.

**Fig. 7:**
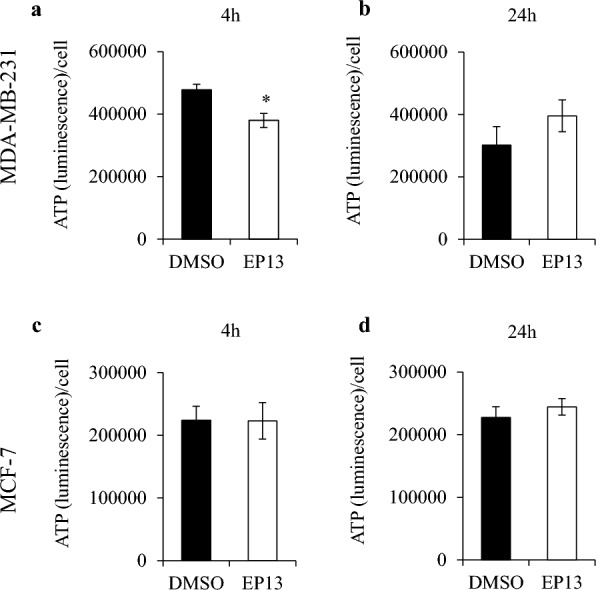
EP13 does not affect ATP levels in MDA-MB-231 and MCF-7 cells. MDA-MB-231 (**a**, **b**) and MCF-7 (**c**, **d**) cells were treated with vehicle or EP13 (6 μM) for 4 and 24 h. Intracellular ATP levels were measured using the CellTiter-Glo® kit and normalized relative to cell numbers. The results are depicted as the mean ± SEM of at least three independent measurements. Significant differences from control cells are indicated (*, p < 0.5)

We then determined the amount of ATP in EP13-treated cells. The cells were treated for 4 and 24 h with EP13, and intracellular ATP was measured by chemiluminescence. Normalization was performed after crystal violet labelling to obtain the number of cells per well. In MDA-MB-231 cells, we measured a lower ATP concentration after 4 h of EP13 exposure in comparison with control cells (Fig. 7a). However, after 24 h, there was no difference in ATP levels between EP13-treated cells and control cells (Fig. 7b). In MCF-7 cells, regardless of the time of analysis, we did not observe any difference between EP13-treated cells and control cells (Fig. 7c and d). These results were in agreement with the hypothesis of a compensatory increase in the glycolytic rate as an adaptation of EP13-treated cells to OXPHOS inhibition.

### EP13 acts synergistically with glycolysis inhibitors to kill breast cancer cells

**Fig. 8:**
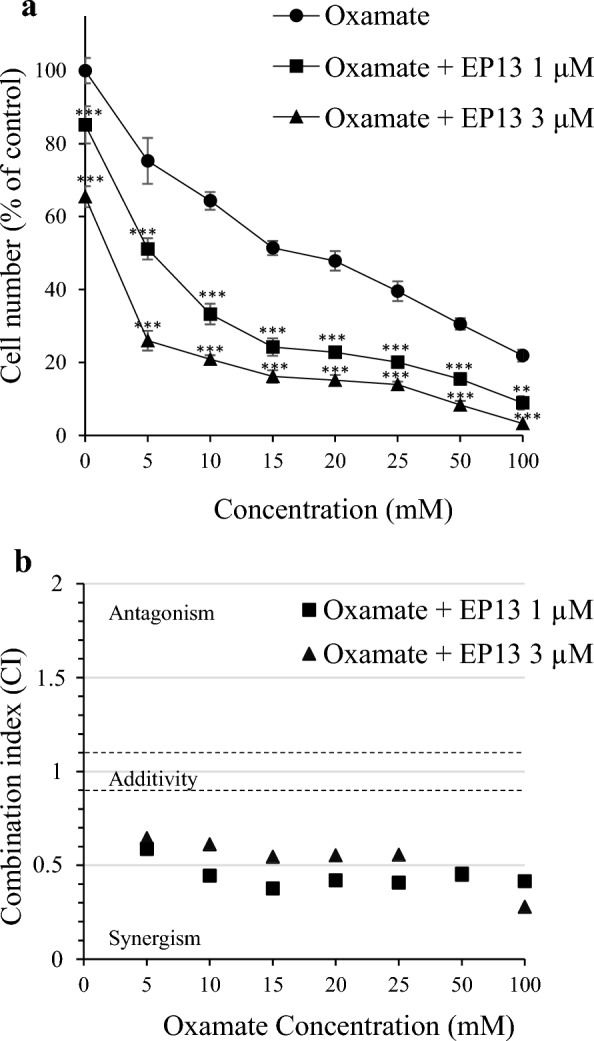
EP13 and oxamate act synergistically to kill breast cancer cells. MDA-MB-231 cells were treated for 72 h with EP13 (1 and 3 µM) and increasing concentrations of oxamate, a glycolytic inhibitor, used alone or in combination. Cell numbers were determined by crystal violet staining (**a**). The results are depicted as the mean ± SEM of at least three independent measurements. Two-way analysis of variance (ANOVA) followed by the Dunnett multiple comparisons posttest were performed. Significant differences from control cells are indicated (**, p < 0.01; ***, p < 0.001). Data were analysed by Compusyn software to determine the combination index (CI) with a nonconstant ratio according to the Chou Talalay method (**b**). The area between the dashed line (0.9 < CI < 1.1) is related to an additive effect. A CI > 1.1 indicates an antagonistic effect, and a CI < 0.9 indicates a synegistic effect

Since EP13 inhibited OXPHOS, we determined whether EP13 could potentiate the anticancer effect of compounds that inhibit glycolysis. First, we used oxamate, which is known to inhibit the activity of the lactate dehydrogenase (LDH-A) enzyme [27]. Oxamate alone induced a dose-dependent decrease in the MDA-MB-231 cell number with an IC_50_ of 18.4 mM. The combination of 1 and 3 µM EP13 with oxamate enhanced the inhibitory effect. Indeed, 51.40% ± 1.96% of the cells were present after 15 mM oxamate treatment alone versus 24.24% ± 2.39% and 16.15% ± 1.17% in combination with 1 µM EP13 and 3 µM EP13, respectively (Fig. 8a). Synergism between EP13 and oxamate was confirmed using the Chou-Talalay CI method, showing CI < 0.9 for all the concentrations tested (Fig. 8b).

**Fig. 9:**
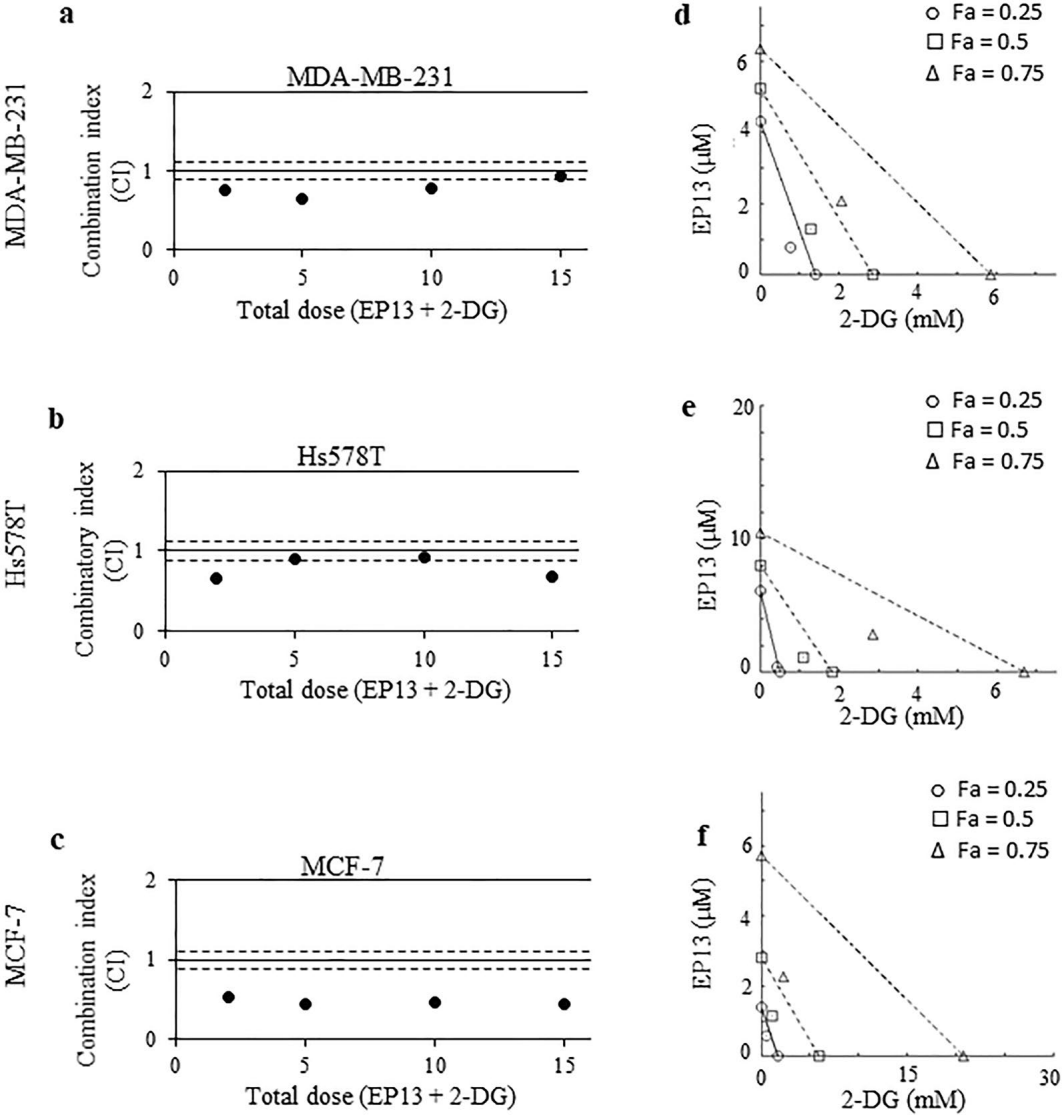
EP13 and 2-DG act synergistically to kill breast cancer cells. MDA-MB-231 (**a**, **d**), Hs578T (**b**, **e**), and MCF-7 (**c**, **f**) cells were treated for 72 h with increasing concentrations of EP13 and 2-DG used alone or in combination at a constant ratio of 1/1. Cell numbers were determined by crystal violet staining. Data were analysed by Compusyn software to determine the combination index (CI) according to the Chou Talalay method (a, b, c). The CI value is given for each total dose corresponding to the addition of the concentration value of EP13 and 2-DG used in combination (a total dose of 2 corresponds to treatment with 1 µM EP13 and 1 mM 2-DG). The area between the dashed lines (0.9 < CI < 1.1) is related to an additive effect. A CI > 1.1 indicates an antagonistic effect, and a CI < 0.9 indicates a synergistic effect. The isobologram analysis shows the effect (Fa: fraction affected corresponding to the cell number decrease (%)) of the combination of the two compounds versus their effect when used alone (**d**, **e**, **f**). The diagonal line joins the individual doses of EP13 and 2-DG on the x- and y-axes to achieve a 25%, 50% or 75% cancer cell number decrease (Fa). The symbols show the dose of each compound used in combination to achieve similar effects. Data points below the line of additivity indicate synergy, and data points above denote antagonism

Then, we used the nonmetabolizable glucose analog 2-deoxyglucose. We observed a synergy between EP13 and 2-deoxyglucose, with CIs ranging from 0.65 to 0.93 in MDA-MB-231 cells, from 0.67 to 0.93 in Hs578T cells and from 0.44 to 0.52 in MCF-7 cells (Fig. 9a–c). Thus, the synergism was stronger in MCF-7 cells. On the isobolograms (Fig. 9d–f) and the dose–response graphs (Additional file 1: Fig. S1), we observed that for a similar efficiency, the dose of the compounds used alone was always higher than the dose of the compounds used in combination.

## Discussion

This study demonstrates that the original troglitazone derivative EP13 inhibits mitochondrial metabolism in breast cancer cells and potentiates the effect of glycolysis inhibitors.

Concerning the bioenergetic profile of TNBC cells (MDA-MB-231 and Hs578T), our data support previous results showing that TNBC cells present a lower OXPHOS than luminal MCF-7 cells [28–30]. Under our conditions, MCF-7 and MDA-MB-231 cells showed similar basal glycolytic activity. However, MDA-MB-231 cells were able to double their glycolytic rate, while MCF-7 cells at the basal level were already at their maximal capacity. Martin and Mc Gee showed that the MCF-7 cell line presents a 70% glycolytic reserve capacity [30]. They also presented a 50% oxidative reserve capacity for MDA-MB-231 cells, in agreement with our data [30]. As previously described, Hs578T cells are quite remarkable because of their high glycolytic metabolism [29, 30]. Hs578T cells use glycolysis and OXPHOS at close to maximal capacity in the basal state. Martin and McGee analysis confirmed the low glycolytic reserve capacity of Hs578T cells but showed a nearly 50% oxidative reserve capacity in their culture conditions [30]. Hs578T cell specificity also relies on the production of ATP, which mainly depends on glycolysis and not on mitochondrial activity, as in the majority of other breast cancer cell lines [30].

It is well described that TZDs exert direct and rapid effects on mitochondrial respiration [31]. For instance, mitochondrial respiration inhibition was observed in a few minutes in response to 10 µM TGZ in T47D breast cancer cells and HCT116 colon cancer cells [32]. In our culture conditions, we did not observe any effect of TGZ on mitochondrial respiration after 4 h of exposure at its IC_50_ (or at 50 µM for Hs578T cells) regardless of the cell line. In contrast, inhibition of mitochondrial respiration was observed in MDA-MB-231 cells after 4 h of exposure to the unsaturated TGZ derivative Δ2-TGZ at its IC_50_. A similar tendency was observed in MCF-7 cells, but there was no effect in Hs578T cells. One might suggest that the lack of effect of Δ2-TGZ in Hs578T was due to the use of a concentration lower than IC_50_ (> 100 µM) [23]. However, compared to the TGZ data, these results suggest that the unsaturation of TGZ improves its mitochondrial inhibition ability. The pyrrolidinedione compound EP13, that is not only an unsaturated but also a desulfurized and deoxygenated derivative of TGZ, induced early OXPHOS inhibition in the three breast cancer cell lines with a higher efficiency than its parent compounds TGZ and Δ2-TGZ. This early event was persistent and still observable after a 24 h treatment in MDA-MB-231 and MCF-7 cells (Additional file 1: Fig. S2a and S2b). Thus, for EP13, there was also a good correlation between OXPHOS inhibition and its anti-cancer effect. [23].

We further validated this result in mammospheres derived from Hs578T cells, with effects observed at a concentration as low as 3 µM. Few data are available on TZDs metabolic activity in 3D culture models. Nevertheless, consistent with our results, Gottfried et al. had previously shown that PGZ (pioglitazone, another TZD derivative) was also able to decrease the oxygen consumption of PC3 and LNCaP spheroids after 2 h of treatment [16].

Among direct intracellular targets of TZDs, TGZ and PGZ have been shown to interact with mitochondrial pyruvate carrier proteins (MPCs) in skeletal muscle cells to inhibit pyruvate-driven oxygen consumption [33]. However, in a hepatocellular model, the direct effects of pioglitazone on hepatocellular pyruvate metabolism were independent of MPCs [34]. Then, we investigated whether EP13 might alter the import of pyruvate in the mitochondrial matrix. Indeed, breast cancer cells were incubated with methyl pyruvate, an analog that freely crosses the outer and inner mitochondrial membranes independently of MPCs. The cells were treated for 48 h with EP13 and methyl pyruvate, and then the number of cells was quantified by crystal violet staining (Additional file 1: Fig. S3). EP13 induced a decrease in the number of MDA-MB-231 and MCF-7 cells. This effect was not abolished in the presence of methyl pyruvate in MDA-MB-231 cells and was even stronger in MCF-7 cells (Additional file 1: Fig. S3). Thus, the anti-cancer effect of EP13 on these breast cancer cell lines did not appear to be a consequence of an inhibition of pyruvate entry into the mitochondria.

The decrease in mitochondrial respiration induced by TZD (TGZ, PGZ, rosiglitazone RGZ) was associated with an alteration of complex I activity of the respiratory chain in rat muscle and liver homogenates and in the human HL60 cell line [35, 36]. Other studies suggest that complexes III and IV could also be targeted by TGZ, PGZ and CGZ [37–39]. EP13 directly targets isolated mitochondria, but this compound inhibits only complex I of the respiratory chain. The early decrease in oxygen consumption observed in the case of EP13 treatment in MDA-MB-231 and MCF-7 cells was associated with an increase in glucose consumption and lactate production, as demonstrated by concentration measurements in conditioned media. We further confirmed the glucose consumption upregulation induced by EP13 in MDA-MB-231 cells by a fluorescent glucose analog 2-NBDG uptake assay (Additional file 1: Fig. S4). After 4 h of treatment, EP13 induced an increase of 19.95% at 3 µM and 27.76% at 6 µM. Similar events were observed after TZDs (TGZ, PGZ, CGZ) treatment in a large panel of cells, including the breast cancer cell lines T47D, MCF-7 and MDA-MB-231 [17, 32]. The glycolytic shift of metabolism probably explains the absence of a significant difference in the cellular content of ATP when measured in mesangial cells treated with TGZ or in the two breast cancer cell lines MDA-MB-231 and MCF-7 treated with EP13 [15]. In other experimental contexts, an ATP decrease was described after TGZ exposure of HepG2 cells, for example [39, 40].

Due to mitochondrial disruption, ROS are often associated with the cytotoxic effects of TZDs [31, 32, 41]. Indeed, the addition of antioxidants (N-acetyl-L-cysteine (NAC) and ebselen) prevented CGZ-induced cell death in glioma C6 and A172 cells [42–44]. EP13 induced a slight increase in ROS production in MDA-MB-231 cells but not significantly in MCF-7 cells. Furthermore, neutralization of ROS by NAC did not restore the effect of EP13 on MDA-MB-231 cells, suggesting that ROS do not contribute to the anti-cancer effect of EP13.

The insulin sensitizer metformin is a biguanide widely prescribed for the treatment of type 2 diabetes mellitus. It has been suggested to function as an antitumoral agent based on clinical data as well as preclinical studies showing an antiproliferative effect in cultured breast cancer cells and animal models [45]. Metformin inhibited mitochondrial respiratory chain complex I, and combined inhibition of the glycolytic pathway led to increased cell death [46–48]. Similarly, Moon et al. showed that reducing glucose availability potentiated the ability of TGZ to reduce T47D breast cancer and HCT116 colon cancer cell proliferation [32]. Moreover, the combination of PGZ with the glycolysis inhibitor 2-DG had an additive effect on the inhibition of proliferation of LNCaP and PC3 prostatic cell-derived spheroids and led to their disintegration [16]. Because convergent data indicate that glycolysis stimulated by metformin, TZDs or EP13 is likely a compensatory response, we hypothesized that combined suppression of glycolysis could enhance the anticancer action of EP13. We first tested the metabolic shift from glycolysis to mitochondrial oxidative metabolism using dichloroacetate (DCA). DCA is an inhibitor of pyruvate dehydrogenase kinase (PDK), and subsequently, it reactivates the pyruvate dehydrogenase (PDH) complex and oxidative phosphorylation (Krebs cycle, respiratory chain and ATP synthesis) along with redox stress [27, 49]. DCA increased metformin-induced oxidative stress and sensitized cancer cells to metformin cytotoxicity [50]. In MDA-MB-231 cells, DCA showed low toxicity up to 25 mM and did not potentiate the anti-cancer effect of EP13 (Additional file 1: Fig. S5). This observation could be the consequence of the absence of ROS in EP13 activity, as previously discussed. To further support the role of glycolysis as a compensatory response to the inhibition of mitochondrial metabolism, EP13 was combined with a known glycolysis inhibitor, oxamate. This compound is described as a lactate dehydrogenase, pyruvate kinase and enolase inhibitor limiting glycolytic flux [27]. In this context, EP13 cytotoxic activity was amplified by oxamate co-treatment in a synergistic way in MDA-MB-231 cells. Similar results were observed in different cancer cell lines when oxamate was combined with phenformin. This antidiabetic agent impaired the oxidative phosphorylation pathway and accelerated lactate production [51]. We then completed our study with a combined treatment of EP13 with 2-DG, another glycolysis inhibitor. 2-DG is a glucose analog that competes with glucose at different levels in the glycolytic pathway. In MDA-MB-231 and MCF-7 cells, the combination of 2-DG and metformin resulted in a marked increase in cell death compared to either agent alone [48]. Treatment with TGZ or PGZ with 2-DG enhanced the antitumour effect of 2-DG in vitro and in vivo [52]. Similarly, the combination of 2-DG and EP13, used at a constant ratio, showed a synergistic effect on the three breast cancer cell lines MDA-MB-231, Hs578T, and MCF-7. The triple-negative cell lines were already responsive to 2-DG treatment alone, and the synergism observed with the combination remained moderate. Higher synergism was observed with the oxidative MCF-7 cells that were the most sensitive to EP13 and the least responsive to 2-DG (Additional file 1: Fig. S1). This result highlights the importance of glycolysis as a compensatory response even in non-glycolytic cells. However, due to the structural similarity of glucose with mannose, 2-DG also competes with mannose for the process of N-linked glycosylation of proteins in the endoplasmic reticulum (ER). Both protein N-glycosylation and glycolysis inhibition contribute to the anticancer effect of 2-DG in MDA-MB-231 and MCF-7 breast cancer cells [53]. Thus, we know that part of the effect of the co-treatment EP13/2-DG is the consequence of N-glycosylation alterations. Overall, convergent data support the role of glycolysis as a compensatory response to EP13 blockade of the mitochondrial respiratory chain.

The pyrrolidinedione EP13, a desulfurized deoxygenated and unsaturated derivative of TGZ, is a potent anticancer compound with activity within a low micromolar range. We demonstrated that EP13 inhibited mitochondrial respiration in the three breast cancer cell lines tested. Compared to TGZ, the structural modifications improved its ability to target the respiratory chain. However, no decrease in ATP levels was detected. Indeed, similar to other TZDs and biguanides, EP13 accelerated aerobic glycolysis and the production of lactate as a compensatory metabolic response. Oxidative stress induced by EP13 was detected in MDA-MB-231 cells, but ROS production did unlikely contribute to the cytotoxic effect of EP13. Our study sheds light on the effects of EP13 on breast cancer cells, but the importance of metabolic impairment in the anti-cancer activity of EP13 has not yet been fully elucidated. Other investigations could be performed to evaluate its potential ability to chemosensitize tumors or to overcome resistance in breast cancer. Inhibition of mitochondrial enzymes by different compounds has been identified as a promising strategy to target cancerous cells [54]. EP13 could be a new member of this family. To optimize its anti-cancer activity, we may consider conjugating EP13 to a triphenylphosphonium moiety. Such a modified molecule, like Mito-metformin, could accumulate in tumor cell mitochondria and efficiently affect cell viability [55]. The combinatorial antitumor effect of mitochondria inhibitors in immuno-oncology is also an exciting perspective.

### Supplementary Information

**Additional file 1: Figure S1.**. EP13 and 2-DG act synergistically to kill breast cancer cells. MDA-MB-231, Hs578T, and MCF-7 cells were treated for 72 h with increasing concentrations of EP13 and 2-DG used alone or in combination at a constant ratio of 1/1. In this last condition, the total dose corresponds to the addition of the concentration value of EP13 and 2-DG used in combination (a total dose of 2 corresponds to treatment with 1 µM EP13 and 1 mM 2-DG). Cell numbers were determined by crystal violet staining. The results are depicted as the mean ± SEM of at least three independent measurements. Significant differences from control cells are indicated. *, p < 0.5; **, p < 0.01; ***, p < 0.001. **Figure S2.** EP13 inhibits mitochondrial respiration after 24 h treatment. MDA-MB-231 (a) and MCF-7 (b) cells were treated for 24 h with vehicle or EP13 (6 μM), and the OCR was measured using an XFe24 Seahorse system. At the times indicated, the following drugs were injected: oligomycin A (Oligo; 2 µM), FCCP (1.1 µM and 2.2 µM), and rotenone / antimycin A (Rot/AA; 1 µM each). Data are the means ± S.D.s (at least n = 3 wells per group). **Figure S3.** Methyl pyruvate does not reverse the EP13 effect in breast cancer cells. MDA-MB-231 (a) and MCF-7 (b) cells were treated for 48 h with EP13 (6 µM) and methyl pyruvate (5 mM). Control cells were exposed to vehicle. Cell numbers were determined by crystal violet staining. The results are depicted as the mean ± SEM of at least three independent measurements. Significant differences from control cells are indicated. ***, p < 0.001; ns, not significant. **Figure S4.** EP13 increases glucose uptake in MDA-MB-231 cells. MDA-MB-231 cells were treated for 4 h with EP13 (3 and 6 µM) or vehicle (DMSO). Glucose uptake was assessed by incorporation of the fluorescent glucose analog 2-NBDG for 1 h in glucose-free DMEM. The median fluorescence intensity was measured and normalized to unstained controls. The protocol is described in Additional file 2. The results are depicted as the mean ± SEM of at least three independent measurements. Significant differences from control cells are indicated. **, p < 0.01. **Figure S5. **EP13 and dichloroacetate (DCA) show additive effects to kill breast cancer cells. MDA-MB-231 cells were treated for 72 h with EP13 (3 and 6 µM) and increasing concentrations of DCA, an inhibitor of pyruvate dehydrogenase kinase (PDK), used alone or in combination. Cell numbers were determined by crystal violet staining (a). The results are depicted as the mean ± SEM of at least three independent measurements. Two-way analysis of variance (ANOVA) followed by the Dunnett multiple comparisons posttest were performed. Significant differences from control cells are indicated. **, p < 0.01; ***, p < 0.001. Data were analysed by Compusyn software to determine the combination index (CI) with a nonconstant ratio according to the Chou Talalay method (b). The area between the dashed line (0.9 < CI < 1.1) is related to an additive effect. A CI > 1.1 indicates an antagonistic effect, and a CI < 0.9 indicates a synegistic effect. The protocol is described in Additional file 2.

**Additional file 2.**. Additional material and methods.

## Data Availability

The datasets used and/or analysed during the current study are available from the corresponding author upon reasonable request.

## Abbreviations

ATP: Adenosine triphosphate
CGZ: Ciglitazone
CI: Combination index
DCA: Dichloroacetate
2-DG: 2-Deoxy-D-glucose
DMEM: Dulbecco’s modified Eagle’s medium
DMSO: Dimethyl sulfoxide
ECAR: Extracellular acidification rate
FCCP: Carbonyl cyanide-p-trifluoromethoxyphenyl-hydrazon
IC_50_: Half maximal inhibitory concentration
NAC: N-acetyl-L-cysteine
2-NBDG: 2-(N-(7-nitrobenz-2-oxa-1,3-diazol-4-yl)amino)-2-deoxyglucose
OCR: Oxygen consumption rate
OXPHOS: Mitochondrial oxidative phosphorylation
PBS: Phosphate-buffered saline
PGZ: Pioglitazone
PPARγ: Peroxisome proliferator-activated receptor gamma
ROS: Reactive oxygen species
TNBC: Triple-negative breast cancer
TGZ: Troglitazone
TZD: Thiazolidinediones
