# Supplementary figures and images for: The troglitazone derivative EP13 disrupts energy metabolism through respiratory chain complex I inhibition in breast cancer cells and potentiates the antiproliferative effect of glycolysis inhibitors
Source: Cancer Cell Int. 2024 Apr 10;24:132. doi: 10.1186/s12935-024-03319-z (PMC11005237; doi:10.1186/s12935-024-03319-z)

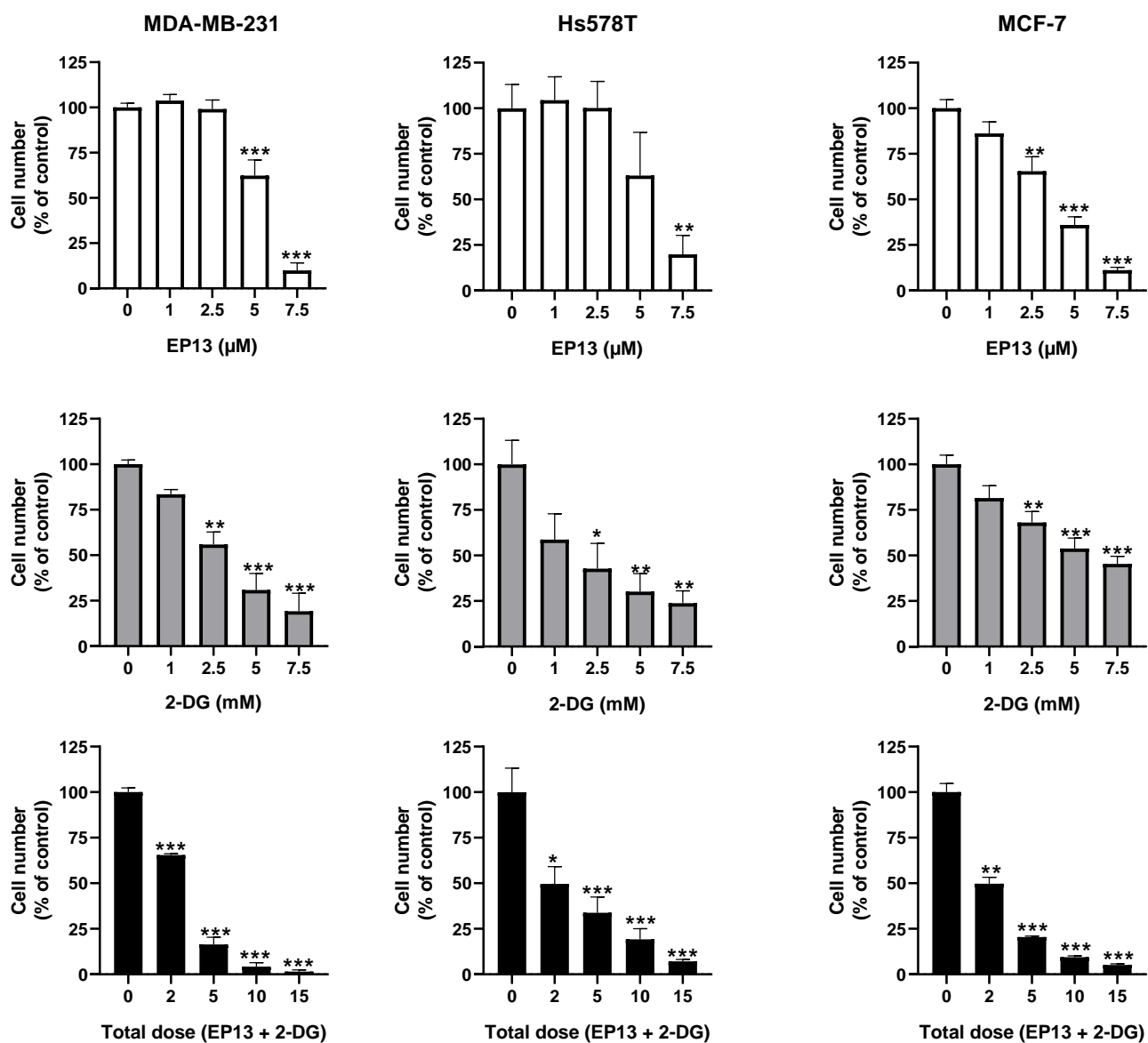

**Figure S1**

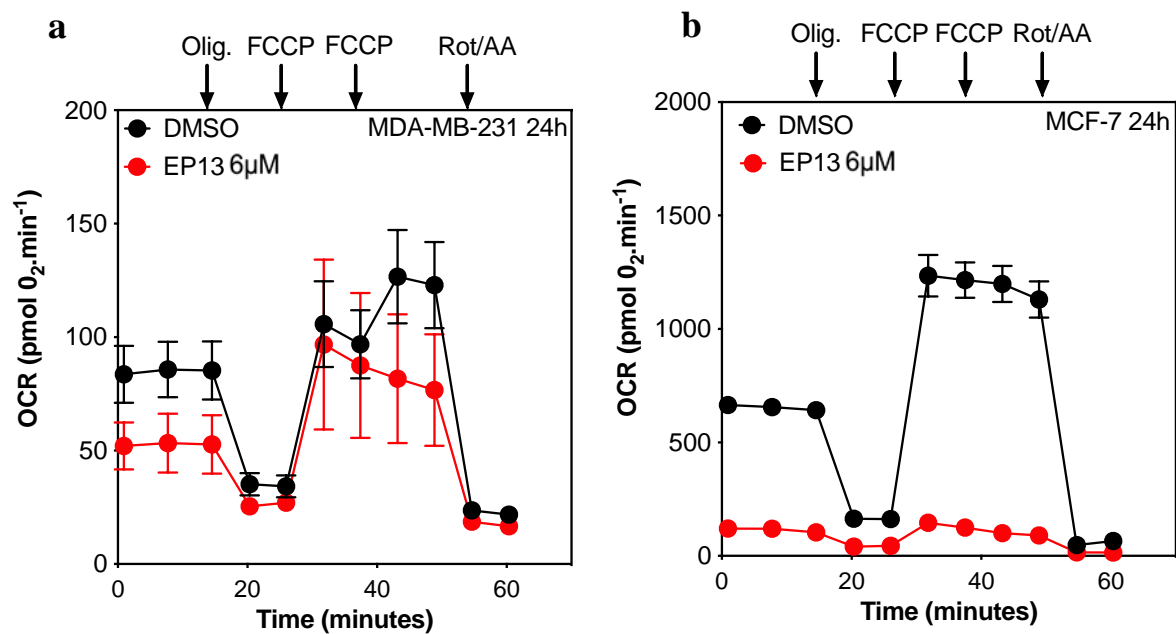

**Figure S2**

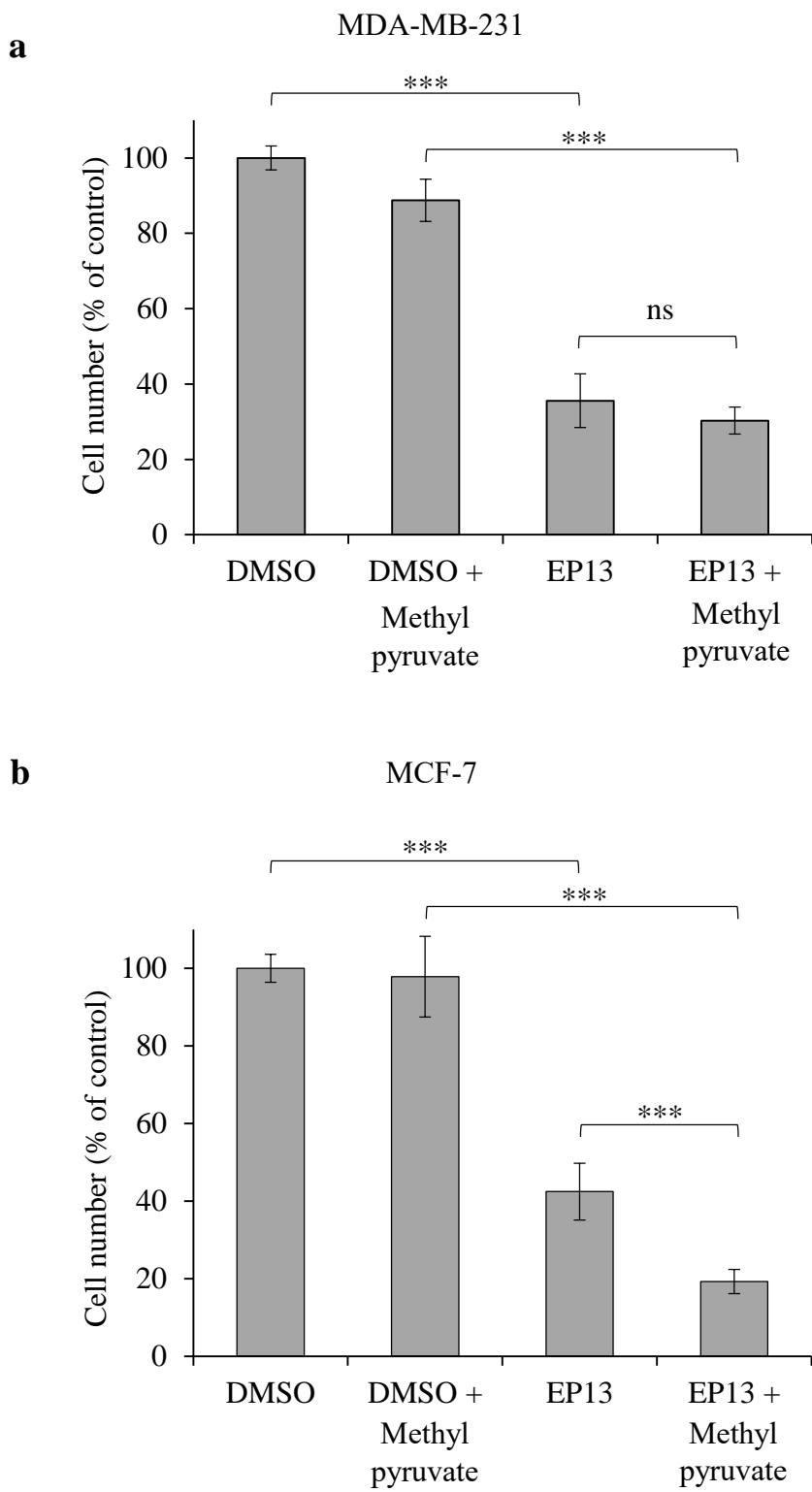

**Figure S3**

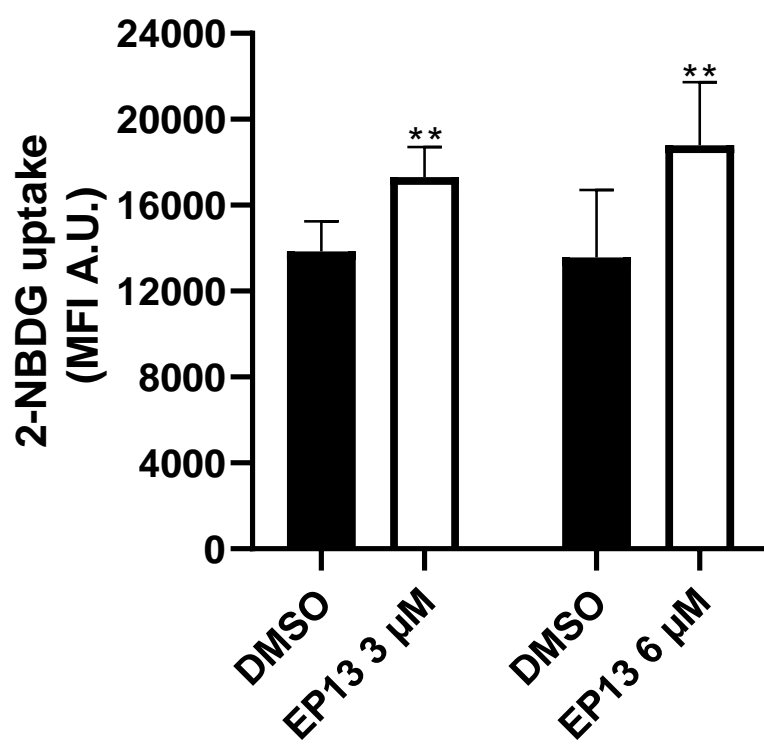

Figure S4

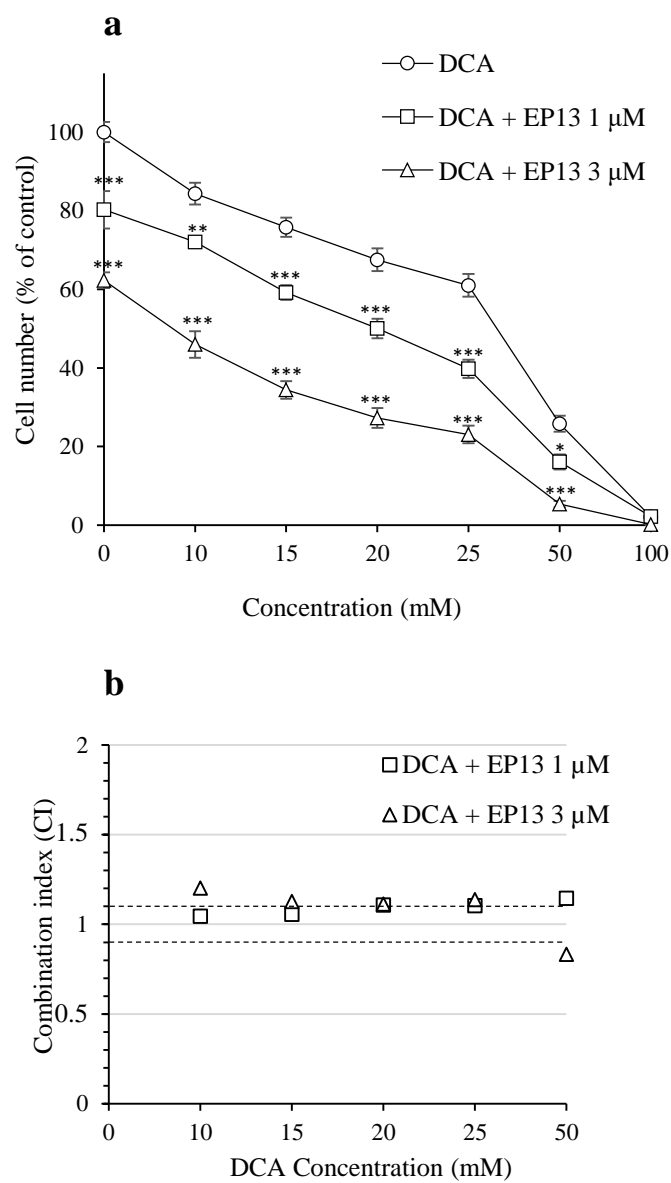

**Figure S5**

Supplement: Supplementary file 1 — Additional file 1: Figure S1.. EP13 and 2-DG act synergistically to kill breast cancer cells. MDA-MB-231, Hs578T, and MCF-7 cells were treated for 72 h with increasing concentrations of EP13 and 2-DG used alone or in combination at a constant ratio of 1/1. In this last condition, the total dose corresponds to the addition of the concentration value of EP13 and 2-DG used in combination (a total dose of 2 corresponds to treatment with 1 µM EP13 and 1 mM 2-DG). Cell numbers were determined by crystal violet staining. The results are depicted as the mean ± SEM of at least three independent measurements. Significant differences from control cells are indicated. *, p < 0.5; **, p < 0.01; ***, p < 0.001. Figure S2. EP13 inhibits mitochondrial respiration after 24 h treatment. MDA-MB-231 (a) and MCF-7 (b) cells were treated for 24 h with vehicle or EP13 (6 μM), and the OCR was measured using an XFe24 Seahorse system. At the times indicated, the following drugs were injected: oligomycin A (Oligo; 2 µM), FCCP (1.1 µM and 2.2 µM), and rotenone / antimycin A (Rot/AA; 1 µM each). Data are the means ± S.D.s (at least n = 3 wells per group). Figure S3. Methyl pyruvate does not reverse the EP13 effect in breast cancer cells. MDA-MB-231 (a) and MCF-7 (b) cells were treated for 48 h with EP13 (6 µM) and methyl pyruvate (5 mM). Control cells were exposed to vehicle. Cell numbers were determined by crystal violet staining. The results are depicted as the mean ± SEM of at least three independent measurements. Significant differences from control cells are indicated. ***, p < 0.001; ns, not significant. Figure S4. EP13 increases glucose uptake in MDA-MB-231 cells. MDA-MB-231 cells were treated for 4 h with EP13 (3 and 6 µM) or vehicle (DMSO). Glucose uptake was assessed by incorporation of the fluorescent glucose analog 2-NBDG for 1 h in glucose-free DMEM. The median fluorescence intensity was measured and normalized to unstained controls. The protocol is described in Additional file 2. [file 12935_2024_3319_MOESM1_ESM.pdf]
