# Supplementary material for: The troglitazone derivative EP13 disrupts energy metabolism through respiratory chain complex I inhibition in breast cancer cells and potentiates the antiproliferative effect of glycolysis inhibitors
Source: Cancer Cell Int. 2024 Apr 10;24:132. doi: 10.1186/s12935-024-03319-z (PMC11005237; doi:10.1186/s12935-024-03319-z)
Supplement: Supplementary file 2 — Additional file 2.. Additional material and methods. [file 12935_2024_3319_MOESM2_ESM.pdf]

Additional file 2: Supplementary material and methods

2-NBDG uptake protocol

MDA-MB-231 cells were plated in 6-well plates at 1.2 x 10^5 cells per well. Twenty-four hours later, the cells were treated for 4 hours with EP13 (3 and 6 µM) or DMSO (control condition). The cells were then incubated in glucose-free DMEM/10 % FCS medium containing 50 µM fluorescently tagged 2-(N-(7-nitrobenz-2-oxa-1,3-diazol-4-yl) amino)-2-deoxyglucose (2-NBDG) (#N13195 Molecular probes, Thermo Fisher Scientific, Illkirch-Graffenstaden, France) for 1 hour before measuring fluorescence by flow cytometry. A total of 10,000 cells were analysed with a CytoFLEX cytometer (Beckman Coulter, France).

Measurement of the combination index after EP13 and DCA cotreatment

Cells were seeded in 96-well plates at 1.5 x 10^4 cells per well. Twenty-four hours later, the cells were treated for 72 h with EP13 (3 or 6 µM) and increasing concentrations of dichloroacetate (DCA), used alone or in combination. At the end of treatment, the number of cells was determined by crystal violet staining assay as previously described [23]. The percentage of cell growth inhibition was then determined for each concentration of compound. Data were analysed using CompuSyn software (CompuSyn Version 1.0 by Ting Chao Chou and Nick Martin, 2004). Combination index (CI) values were determined to assess the nature of compound-compound interactions that can be additive ($0.9 < CI < 1$), synegistic ($CI < 0.9$), or antagonistic ($CI > 1.1$) for various concentrations.
